# A Critical Perspective on Neural Mechanisms in Cognitive Neuroscience: Towards Unification

**DOI:** 10.1177/17456916231191744

**Published:** 2023-08-29

**Authors:** Sander van Bree

**Affiliations:** 1 Centre for Cognitive Neuroimaging, School of Psychology and Neuroscience, University of Glasgow; 2 Centre for Human Brain Health, School of Psychology, University of Birmingham

**Keywords:** neural mechanism, Marr’s three-level framework, theoretical unification, randomness, levels of scale, effect size, cognitive neuroscience, new mechanical philosophy, philosophy of science

## Abstract

A central pursuit of cognitive neuroscience is to find neural mechanisms of cognition, with research programs favoring different strategies to look for them. But what is a neural mechanism, and how do we know we have captured them? Here I answer these questions through a framework that integrates Marr’s levels with philosophical work on mechanism. From this, the following goal emerges: What needs to be explained are the computations of cognition, with explanation itself given by mechanism—composed of algorithms and parts of the brain that realize them. This reveals a delineation within cognitive neuroscience research. In the *premechanism stage*, the computations of cognition are linked to phenomena in the brain, narrowing down where and when mechanisms are situated in space and time. In the *mechanism stage*, it is established how computation emerges from organized interactions between parts—filling the premechanistic mold. I explain why a shift toward mechanistic modeling helps us meet our aims while outlining a road map for doing so. Finally, I argue that the explanatory scope of neural mechanisms can be approximated by effect sizes collected across studies, not just conceptual analysis. Together, these points synthesize a mechanistic agenda that allows subfields to connect at the level of theory.

Cognitive neuroscience has emerged as a science that aims to explain how cognition is implemented by the brain. There is a sense across much of the literature that this project involves more than just accurately predicting brain responses or the behavior they produce. Although prediction may aid in developing an explanation or testing the validity of an explanation, and although it serves a plethora of clinical and pragmatic ends, explanation ultimately requires the specification of *neural mechanisms*.

## Introduction

Although the specification of neural mechanisms is widely accepted as a principal goal in cognitive neuroscience, what is understood by the term varies significantly across the scientific literature. For one, the term *mechanism* is used to describe parts of the brain across levels of size, from neurotransmitters to anatomical brain regions. Second, the term is used when complex rules govern how these parts interact and when they simply activate in a fixed sequence. Third, the term is used when correlations between the brain and cognition have been found and when such relations are proved causal. Fourth, the term is used when parts of the brain have small effects on cognition and when they explain much of the variance. Which of these are neural mechanisms of cognition, which are not, and why does it matter?

The aim of this contribution is to start with a robust conception of *mechanism* and to analyze how it fares in cognitive neuroscience—with the intention of promoting a mechanistic agenda that applies broadly. To do so, I will first introduce prominent research programs, contextualizing them in Marr’s three-level framework (Marr, 1982). Then, I will review two examples of explained information-processing systems outside of neuroscience, where the mechanisms have been extensively described. From this vantage point, I will distinguish two stages of research in cognitive neuroscience: the stage at which context is given to mechanism, and the stage at which mechanism itself is explained. I argue that research in the former stage is often inaccurately considered to specify mechanism itself, that this hampers theoretical unification, and that characterizing mechanism itself should be given more focus. I will also discuss an example of a modeled neural mechanism of cognition, highlighting what sets it apart from nonmechanistic models. Finally, I show criteria that mechanism does not necessarily need to meet, and why, beyond establishing mechanism itself, it is important to estimate the explanatory scope of a proposed mechanism using effect sizes collected across data.

## How We Look for Mechanisms in Neuroscience

Historically, Marr’s three-level framework (Marr, 1982) has proven an invaluable beacon in the search for neural mechanisms of cognition. Its central tenet is that the brain—like any information-processing system—requires an account of three levels. At the top, the *computational level* describes what is being computed and why—requiring at minimum a specification of the input–output function that is being computed.^1^ This level details what it is that needs mechanistic explanation. Mechanism itself is spelled out by the remaining levels (Craver, 2007). Specifically, the *algorithmic level* describes the representations of the system and the transformations that are performed over it, and the *hardware-implementation level* lays out the physical parts that implement these algorithms, such as molecules, cells, synapses, or networks. A successful explanation in neuroscience is one that marries these levels, such that the physical parts realize algorithms, which in turn realize computation (Marr, 1982).

**Fig. 1.**
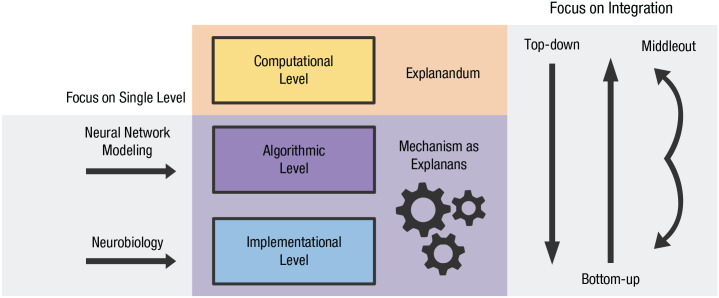
Programmatic approaches to explaining cognition in the brain. In cognitive neuroscience, the *explanandum*—that which is to be explained—is the computation of cognition. The *explanans*—that which does the explaining—is spelled out by mechanism itself, which demands description at the level of algorithm and implementation. The fields of artificial neural network modeling and neurobiology reveal details about algorithm and implementation, respectively. And top-down, bottom-up, and middle-out approaches programmatically set out to integrate Marr’s levels, each pursuing a different order of priority in light of metatheoretical considerations.

Although there is widespread—but not universal (Buzsáki, 2019; Churchland, 1986)—agreement on the value of Marr’s framework, there is ample disagreement as to how research programs should be designed to meet its demands (Fig. 1). Subfields have proclivities toward different levels of analysis, each championing its own flavor of scientific explanation. The field of artificial neural network modeling—also called *neuroconnectionism* (Doerig et al., 2023)—abstracts away from neurobiological detail to figure out how the brain essentially computes, which as a result also elucidates the algorithms that underpin neural processing (Lindsay, 2021). The field of neurobiology instead concentrates on hardware implementation, with a stronger emphasis on the physiological details of biological entities and the role they play in the brain.

Then there are programs that expressly aim for integration across levels, with different programs varying in their preferred order of priority of Marr’s levels. In a bottom-up approach, biology constrains algorithm, which in turn constrains computation. This approach maintains that we should understand the brain by first mapping out its parts before we can get a handle on the algorithms those parts realize to perform the computations of our cognitive repertoire (Braun, 1990). Advocates of this approach argue that starting with neural data minimizes reliance on a cognitive ontology—by letting the brain speak for itself, we sidestep entrenched and potentially wrong assumptions about the mind (Buzsáki, 2019). In a top-down approach, the order of constraint is reversed. Here, emphasis is placed on abstractions of cognitive function, with our computational repertoire adjudicating between different architectures before turning to where in the brain these architectures might reside (Marr, 1982; van Rooij & Baggio, 2021). Some have argued for this approach on the basis that pursuing an explanation before specifying a target capacity is like searching for answers before posing a question; in other words, to adequately study how the mind is physically implemented, we must first characterize it (Poeppel & Adolfi, 2020). Finally, a middle-out approach is gaining traction, with the algorithmic level constraining both of its neighbors. Here, neural mechanisms are obtained by validating competing algorithmic accounts of cognition on neuroimaging data while separately branching out to the computational level (Love, 2015). This approach has the advantage of aiming from the outset to factor in constraints set by different levels. Together, these approaches make up the prominent strategies for acquiring mechanistic understanding of cognition in the brain.

But what does mechanistic understanding itself consist of?

## Mechanism Is a High Bar to Clear

A mechanism for any phenomenon is defined by entities whose activities and interactions are organized in such a way that they produce that phenomenon (Glennan, 1996). This definition, which is similar to other accounts (Craver, 2001, 2007; Wimsatt, 1997), ties into Marr’s framework in the following way. The phenomenon produced by the mechanism is described by the computational level, making up the explanandum. The explanans is given by the mechanism itself, which can be described both in algorithmic terms (i.e., by rules that operate upon input representations to enable computation) and in physical terms (e.g., by the neurons and synapses that realize the algorithms).^2^

Marr’s framework and mechanistic philosophy remind us that, contrary to what is sometimes believed in neuroscience, mechanism is not just described by the set of physical parts that underlie a cognitive function—the implementational level. The brain also has design principles that govern how physical parts interact to compute—and this is provided by the algorithmic level (Bechtel & Shagrir, 2015). These design principles are abstract: They detach conceptually from the specific parts out in the world that happen to realize them (Levy & Bechtel, 2013). Crucially, then, to describe a neural mechanism of cognition, a formulation of either the implementational or algorithmic level alone does not suffice. An inventory of neurons and their synapses does not describe a mechanism in neuroscience, and neither does a computational model that floats free from neural detail. For a neural mechanism of cognition to be described, both levels must be supplied.

Moreover, even when both levels are supplied, the posited hardware candidate must be able to realize the algorithms, and must do so in the way the brain does, separating *how-actually* from *how-possibly* explanations (Craver, 2006; Guest & Martin, 2023). For example, a circuit of cells that implements behavior using algorithms different from those of the brain is not a neural mechanism of cognition, and neither is a behavior-approximating algorithm that can only be implemented using gears.

A description that adequately captures Marr’s levels—a *Marrian description*—basks in conceptual clarity. Here, one can freely move between levels, defining algorithms in terms of physical parts and vice versa. An external observer of a Marr-described system could look at the system’s physical parts and use an instruction manual that lists the algorithms to approximate the system’s resulting computations. Trivially, there might be epistemic limitations standing in the way of such an analysis—such as the inability to observe system parts, to calculate a state transition, or to keep track of system states. But there is no conceptual vagueness as to how computation emerges from mechanism, and so there is no problem in principle, even if there may be one in practice.

## Examples of Described Mechanisms

It may be instructive to call on two paradigmatic examples of Marr-described information-processing systems in which both the computations and the mechanisms that realize them have been richly described. These are random access memory, or RAM, and DNA, which are both examples of subsystems embedded in a larger system. Each of these subsystems implements the minimal computation of memory: information storage for future retrieval.

RAM can be described on the algorithmic level as achieving computation by routing inputs to memory addresses that have information stored in the form of binary symbols, which can be extracted (read) and changed (write) before the system moves to a new state, at which point the same logic repeats. On the implementational level, we can describe the parts that realize these abstract design principles: The symbols are physically realized by transistors on a silicon wafer, and their value can flip via electric signals that switch the transistor between “on” and “off” states.

DNA has an algorithmic account similar to that of RAM’s read component—here too are symbols stored, extracted, and manipulated according to rules. On the implementational level, the physical parts are made of carbon rather than silicon, with the symbols realized by nucleotide bases of DNA strands—adenine, cytosine, guanine, and thymine—which variously combine to code the proteins necessary for life.

For these two memory systems, we have a good sense of how computation arises because we have extensively mapped out the mechanisms in terms of their algorithms and the physical parts that realize them. As a result, we can wield our mechanistic understanding of these systems in powerful and creative ways, such as to predict disease and explain biological phenomena in the case of DNA, or to build computers and phones with ever-increasing storage capacity in the case of RAM.^3^ With that said, a complete explanation requires more detail than this section can provide, such as how these subsystems are situated within the overall architecture and how they interact with other subsystems (a point that extends to mechanisms in the brain). Nevertheless, these two well-specified systems offer an intuitive way to think about mechanistic explanation.

## Mechanistic Understanding

In assessing whether a mechanism has been understood, we cannot rely on a subjective sense of insight because this experience is known to be highly fallible (Danek & Wiley, 2017). A more objective way to evaluate understanding is to ask whether we have “what-if-things-had-been-different” style answers (Craver, 2006; Woodward, 2003). Under this conception, to understand a system is to know what would happen to the explanandum (computation) if the factors in the explanans (mechanistic processes) varied across a wide range of circumstances (Woodward, 2003, p. 11). How should we evaluate whether we have such knowledge?

The approach favored here is to leverage mechanistic descriptions to programmatically alter computation through interventions in the system. If it is true that we know how the physical parts interact to result in a certain computation or other, then we should be able to intervene on the system’s parts in such a way that output B obtains rather than output A. The upshot of this intervention-heavy approach to understanding is that it establishes whether the mechanisms we postulate are actually causing the computations of interest, thereby validating a necessary property of any mechanism. With that said, in cases where perturbing a system is not feasible (for technical reasons) or undesirable (for ethical reasons), an alternative is to ask whether we can use the mechanistic descriptions to predict computation without impinging on the system itself. Under this route, we look at the physical parts of the mechanism, and, using our formulation of the rules by which they interact to implement algorithms, we ask if we are able to approximate how a certain input will result in a certain output. And at intermediary stages of computation, where input representations have already undergone various transformations, we should be able to look at physical parts of the mechanism and approximate how the system will continue on to transform the representations into a certain output.

Importantly, using prediction in this way is different from predictions based on brute-force statistical regularities (Katz, 2012)—an approach that is more common in neuroscientific practice (more on this later). The difference is that in the approach defended here, prediction is methodically confined to a mechanistic explanans itself rather than to any detectable correlation in the system which may or may not be mechanistically relevant (Craver, 2006). Confining prediction in this way is important because it separates real and illusory understanding. For example, if we want to evaluate whether we understand how a car manages to drive at a certain speed, the critical question is whether we can make predictions based on parts that are actually relevant in getting the car to drive. By contrast, if we make predictions based on parts of the car that are not included in the mechanisms for driving, such as the pointer on a speedometer, then we may get highly accurate predictions, but that result is no indication that we understand the system.

If we contextualize these points within the longstanding debate on whether prediction or explanation is most conducive to scientific insight (Yarkoni & Westfall, 2017), then this proposal falls in the explanation camp—yet it rejects a rigid dichotomy by taking on board prediction as an evaluative tool. Together, what emerges is a picture in which understanding manifests as experimental control and predictive power over a system’s behavior based selectively on the mechanisms we put forth. In the upcoming sections, I will contrast this notion of mechanism and scientific understanding with how we think about these concepts in cognitive neuroscience.

## Usage of Mechanism in Neuroscience

In cognitive neuroscience, the term *mechanism* is often invoked when merely a relation between a phenomenon in the brain and cognition has been observed. The phenomenon might involve specific parts of the brain—such as neurotransmitters, neurons, circuits, networks, or anatomical regions (Bassett & Sporns, 2017)—or it might involve a class of brain dynamics, such as event-related potentials (ERPs), traveling waves, or neural oscillations. Moreover, the brain-to-cognition relation might be found on the basis of correlational studies (Mehler & Kording, 2020) or through causal intervention (Dijkstra & de Bruin, 2016).

But although brain-to-cognition relations can be useful, mechanism itself is not described until there is a detailed account of how specific parts of the brain systematically interact to produce cognition (Barsalou, 2017; Hommel, 2020; Krakauer et al., 2017). Even if a brain-to-cognition relation proves causal, by itself it does not describe a neural mechanism because there is no explanation as to how specific physical parts in the system interact to implement algorithms to perform the computations of cognition. To flesh out these points, let us review how cognitive neuroscience research usually transpires and what kinds of explanations are provided by its various stages.

## Stages of Cognitive Neuroscience Research

Cognitive neuroscience typically undergoes several stages of research. Usually, we start with the search for relations between neural phenomena on the one hand (such as brain regions, networks, oscillations, or ERPs) and the various subcomputations of cognition on the other (such as memory encoding, storage, and retrieval)—seeking brain-to-cognition relations. Then, we may test whether these relations are causal while at the same time supplying them with spatial and temporal detail. On top of that, we may describe how different brain-to-cognition relations are linked together, detailing how cognitive processing unfolds in the brain.

**Fig. 2.**
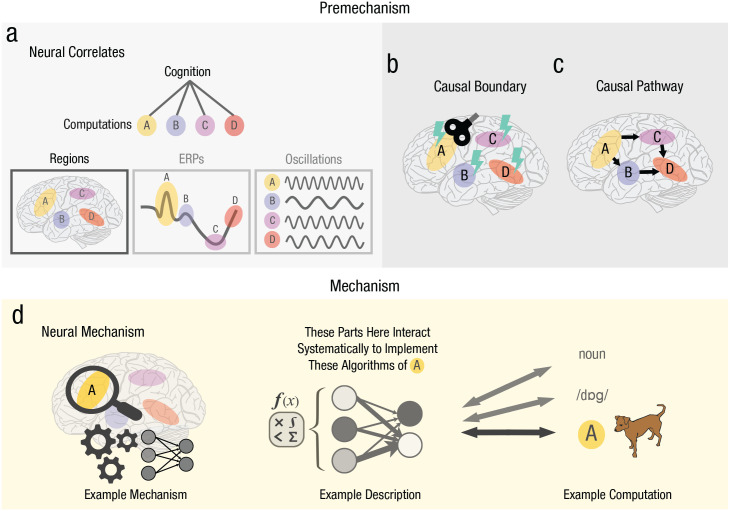
Stages of research in explaining cognition in the brain. Neural correlates stage (a), Cognitive functions can be decomposed into computations that correlate with phenomena in the brain—such as a set of anatomical regions, event-related potentials (ERPs), or frequency bands (or phases) of oscillations. At the causal boundary stage (b), causal studies can determine the boundaries of cognition in the brain, limiting where and when mechanism resides. This stage may confirm, falsify, or adjust the overall model from the neural correlates stage. By the causal pathway stage (c), a preliminary pathway of cognition may have been sketched by means of neuroimaging and brain stimulation. In this stage, however, this sketch is scrutinized and adapted by means of, for example, time-resolved brain simulation, revealing the causal flow of computation. We address neural mechanism itself (d), only when we outline how specific computations emerge from organized interactions between specific parts of the brain. This stage fills the mold of our causal pathways with mechanistic detail, offering *what-if-things-had-been-different* answers on cognition in the brain. Note that the order presented here may vary within research programs (across work on different cognitive functions) and between programs, and stages may sometimes be skipped altogether or returned to later.

These stages of research—which may not be undertaken in the same order—provide premechanistic understanding (Fig. 2). They establish with increasing certainty where and when neural mechanisms of cognition are to be found in the brain. In the neural correlates stage, we specify what phenomenon it is in the brain that relates to cognition (Fig. 2a). In the causal boundary stage, we may use causal interventions to figure out the physical perimeters of cognition, establishing where in space and time mechanism likely^4^ resides (Fig. 2b). In addition, by specifying the flow of computation across anatomical regions (or other neural phenomena), we obtain a causal pathway of cognition (Fig. 2c).

Critically, though, at none of these stages do we explain a neural mechanism of cognition. What is missing is a formulation of how parts in anatomical regions (or networks, ERPs, or oscillations) realize computation (Fig. 2d). What are the ways in which parts of the brain—such as a group of connected neurons—interact, and how does this lead to one computation or another? Can we look at the brain and apply a rulebook with algorithms to physical parts situated in the causal boundary to make any sort of prediction about the computation at hand? If the answer is no, then the stage of research is premechanistic.

Let us take models of language processing to make these points more concrete (see Friederici, 2011, for an overview)—though the argument holds for cognitive neuroscience research at large. At the neural correlates stage, a set of four anatomical regions might be found to correlate, in turn, with phonological, lexical, syntactical, and conceptual processing (these are examples of psycholinguistic computations). Next, when brain stimulation is applied to each anatomical region, it may be found that each region’s computation is impaired without affecting the other regions’ computation except by altering their input,^5^ sketching the causal boundaries of cognition. Then, by taking neuroimaging findings on the temporal dynamics of neural activity across these regions, and by further testing exactly when brain stimulation interferes with different regions, the model can be enriched with the flow of computation, obtaining a causal pathway of cognition.

But a causal pathway of cognition is not a neural mechanism of cognition (Ross, 2021). Pathways detail how information flows through the brain, but they do not address by what parts and methodology information is generated or transformed. Rather, for this model of language processing to address mechanism, there needs to be a formulation of how the physical parts of implicated regions systematically combine to perform one computation or another. How is it that the neurons and synapses in the brain regions stipulated to process the phonological, lexical, syntactical, and conceptual content—say, of the word “dog”—interact to do so (Gallistel & King, 2009; Poeppel, 2012)? What are roughly the rules of interaction by which one computation obtains rather than another (such as the linguistic content associated with “cat”)? Undoubtedly, a complete answer to these questions is difficult to achieve. But my point is that this is a separate class of inquiry that is about mechanism itself, rather than about where in the brain the mechanism resides, the time at which it does its job, its slot in a sequence of multiple mechanisms, or any other context that can be given about it (Hommel, 2020).

A strict emphasis on the conjunction between “how” (algorithm) and “in which specific parts” (implementation) tends to diverge with the usage of the term “mechanism” in cognitive neuroscience, where a correlational or causal relation between regions, ERPs, or oscillations and cognition often suffices (for a few examples in language processing, see Dröge et al., 2016; McNealy et al., 2006; Rodd et al., 2005; Zimmerer et al., 2019). Perhaps it is more appropriate to call a series of correlational findings “neural correlates” rather than “neural mechanisms of cognition” and to refer to findings in the causal realm as “causal boundaries,” or “causal pathways” if the flow of computation is specified.

Regardless of language use itself, the more pertinent point is that mechanistic explanation is distinct from collating a series of correlational or causal findings and supplying them with spatial and temporal detail. Mechanistic explanation consists in describing algorithms and the physical parts of the brain that realize them, making it possible to programmatically alter computation or to predict it across a variety of contexts (such as input stimuli). Seeing this as a distinct enterprise from premechanistic work is important to ensure that research programs can ultimately reconcile their findings at the level of theory and scientific explanation. In this sense, mechanism establishes a universal language to a pluralistic cognitive neuroscience.

## Mechanistic Models in Neuroscience

Up to this point, I have focused on examples of the premechanism stage in cognitive neuroscience, which is differentiated from the mechanism stage proper. For research to be situated in the mechanism stage, two minimal requirements must be met. First, there needs to be some account of the algorithms that govern how representations are transformed, where algorithms are defined as sequences of operations that manipulate one or more variables—analogous to instructions in the form of computer code. Such operations can be described in mathematical terms (like summation, integration, and thresholding), but they need not be. Rather, what is essentially required is a specification of the rules that work over system inputs, and both formal and natural language can achieve this. The second requirement is that any algorithmic specification must go together with empirical data that points to specific parts of the brain that implement the representational transformations, lifting the cognitive model into the neural realm. What do models of this sort look like?

**Fig. 3.**
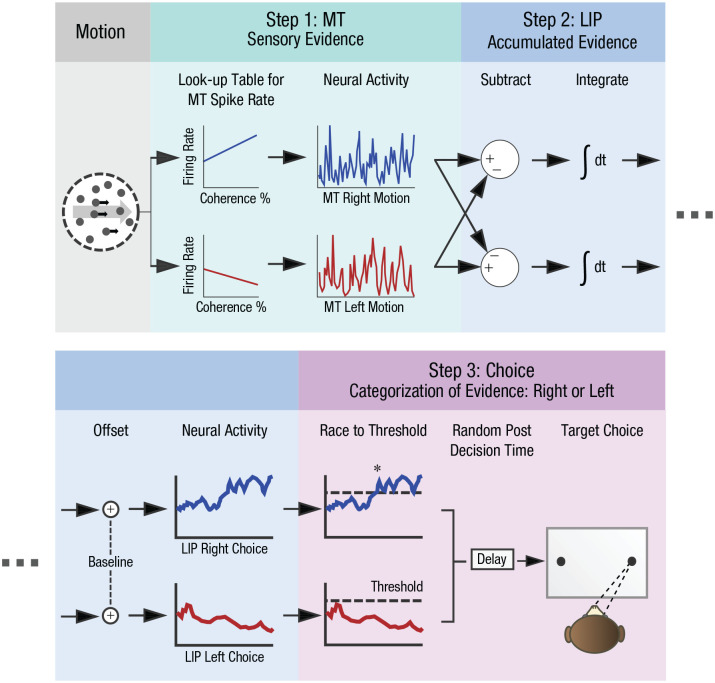
A mechanistic model of visual decision-making in the brain. Fixating monkeys were presented with random motion at a specific strength (coherence percentage) and direction. The animals indicated their decision on motion direction using an eye movement. A model specified the putative neural mechanisms behind this process, containing rules that operate over incoming neural activity (algorithm), as well as ensembles of specific anatomical regions that realize them (implementation). The model accurately predicted decisions, reaction time, and neural activity of empirical data using three steps (Mazurek et al., 2003). First, direction-selective neurons in extrastriate area MT code sensory evidence about motion direction. Second, the difference in activity between left- and right-selective MT cells is integrated and baseline-offset in the LIP. These operations cause left and right-choice LIP neurons to increase or decrease their activity over time as a function of motion direction. Third, a threshold determines when enough evidence has accumulated to categorize evidence as leftward or rightward, initiating the relevant motor programs to make an eye movement toward a target. The asterik (*) represents activity that surpassed the threshold. Redrawn from Mazurek et al. (2003). MT = middle temporal area.

To highlight one comparatively well-specified account, Mazurek et al. (2003) laid out a primate model of visual decision-making (Fig. 3). Here, just as in premechanistic accounts, there is a sequence of brain regions with associated computations, covering each step from stimulus processing to a decision on motion direction. What elevates this account into the mechanism stage is that there is a formulation of the algorithms that achieve the system’s representational transformations, with specific parts empirically validated to implement these algorithms. First, the sequence of computations is explained through a series of operations such as subtraction, integration, relative offsetting, and the dynamic accumulation of evidence up to a threshold. Each of these operations acts upon representations—either those fed into the mechanism, or those derived along the way. Second, the specified algorithms are verified to be realized by neuronal groups in the middle temporal area (MT) and in lateral intraparietal cortex (LIP)—specifically, their activity patterns unfold in concordance with the mechanistic model.

The previously discussed metric for mechanistic understanding goes a long way here—it is through our understanding of how specific parts of the brain interact that prediction on the resulting motion direction decision is made possible. An external observer (in practice, a model wielding our mechanistic hypotheses) could look at stimulus input patterns or neural activity midway and predict—using an explanans—which decision a primate will make on the question of motion direction. Indeed, the authors used their model to successfully predict behavioral and neural measurements (Mazurek et al., 2003). Furthermore, because the algorithms are tied to specific parts of the brain, specific hypotheses emerge on how to manipulate the brain to change the likelihood that one or another target choice obtains, as I will highlight in a fictional experiment later on. In a nutshell, this model exceeds premechanistic explanation because it allows us to predict and control computation by harnessing algorithms that operate in empirically linked parts.^6^

There are two points worth emphasizing at this intersection. First, in our evaluation of the model, prediction is considered only a means to an end—again, it serves as a tool to test and construct an explanans. Indeed, applying pattern classifiers or related statistical techniques to the data will probably yield more accurate predictions about decision-making and neural activity than a model that confines itself to mechanistic parts. However, as with speedometer-based prediction, the claim that this makes such techniques superior to mechanistic models is conflating an instrument for explanation with scientific explanation itself (van Rooij, 2022). Techniques geared to maximize predictions by indiscriminately capitalizing on any pattern in the data are both common and of limited use in neuroscience—they can, for example, help narrow down where computation happens in the brain and thereby bootstrap the process of explanation-building. However, prediction should not be confused with mechanistic understanding (Cummins, 1995, 2000; Kaplan, 2011).

A second point is that the mechanistic model discussed is of a specific kind: It takes neurons and their synaptic connections to be the primary implementer of computation—that is, these physical aspects of the system are granted the most explanatory importance. The field is undergoing a debate on whether this kind of view is right or whether neural computation is distributed to such an extent that a dynamical-systems perspective is required in which the primary implementer is taken to be the neural activity across a population of neurons considered en masse, which undergoes trajectories in multidimensional state space as it realizes computation (Barack & Krakauer, 2021). It is important to delineate strong and weak views of this sort. Strong dynamical systems accounts consider trajectories in state space and the manifolds embedded within them as not just descriptions, but as full-fledged explanations that nullify or firmly relegate the need to identify the specific parts that realize the observed activity patterns, thus forwarding a form of nonmechanistic explanation that is incompatible with the perspective laid out here (Chemero & Silberstein, 2008; Cosmelli et al., 2007; see also van Gelder, 1995). On the other hand, weak views do not take such mathematical descriptions to rise to the level of explanation. Instead, they cast them as instruments that elegantly capture large swathes of interacting parts with the potential to reveal details about a mechanism’s organizational features (Gardner et al., 2022; Langdon et al., 2023). Seen in this light, state space descriptions can guide a mechanistic neuroscience that ultimately seeks to identify parts of the brain that implement the algorithms of computation by offering a springboard for experimentation and computational probing, or even by helping to uncover mechanistic parts directly (Valente et al., 2022). Such a perspective is compatible even with strong accounts in mechanistic philosophy (Kaplan & Craver, 2011).

Following this brief detour, I will next take my critique as it has been developed thus far and complement it with some prescriptions for the field.

## Mechanism Is Not an Out-of-Reach Stage That Needs to Wait

As I have discussed, cognitive neuroscience tends to concentrate on characterizing brain-to-cognition relations, with descriptions of mechanism itself as a separate stage that usually comes later or not at all. There are two normative claims I would like to offer: First, we should recognize this distinction in our work to facilitate unification across fields in the universal language of mechanism, conceiving of the mechanism stage as a distinct end goal. Second, we should expend more resources on the mechanism stage itself. Currently, most efforts are dedicated to providing ever more background detail, as opposed to filling out the premechanistic molds we already have. But if mechanistic explanation is a central goal of cognitive neuroscience, this balance should shift. In Box 1, I outline some recommendations that serve to assist the project of mechanistic modeling.

A possible response to the claim that we should refocus our efforts is to argue that we first need to fully flesh out the context of mechanism before we can sensibly turn to mechanism itself. Put differently, perhaps completing the premechanism stage is simply necessary before we can even start to think meaningfully about mechanism. This would, in turn, suggest that cognitive neuroscience is on the right track in its primary focus on detailing brain-to-cognition relations.

In reply to this, it may be helpful to first reiterate what premechanistic research gets us and what it does not. What findings in this stage offer is an upper bound of when and where mechanisms operate. But neural correlates, causal boundaries, and causal pathways do not address what algorithms are at play, nor do they describe the exact parts that do the job of implementing them. This is evidenced by the fact that a model that employs only these findings cannot explain across a wide range of circumstances (such as sensory inputs) why one or an other computation obtains (such as the linguistic content of “cat” vs. “dog,” a left vs. right target choice, or an oval vs. round face representation). Rather, the honeypot of what-if-things-had-been-different understanding is offered by mechanistic models. Next, I argue that such models can be productively worked on even with only approximate spatial and temporal boundaries (see also Box 1).

**Box 1.** A Road Map for Mechanistic Modeling

Proceeding from varying levels of insight on where and when mechanisms reside in the brain, researchers may wish to focus on mechanistic model development. In this box, I offer three considerations that enable this pursuit, taking the cognitive function of face processing as a running example. **Clarification of the explanandum** By clearing up what it is that we are trying to explain, we can narrow down what a mechanistic model that puts forth the explanation needs to look like. This can be done in different ways. Most formally, we can perform computational complexity analysis to find mathematical and logical constraints on the nature of cognitive computations themselves—thereby also constraining what algorithms could be at play in the explanans (van Rooij & Baggio, 2021). Less formally, we can clarify the explanandum by characterizing in explicit terms what computations we are trying to explain. Is our model attempting to address why in some cases a round face is perceived, and why in other cases an oval one is perceived? Or is the question rather why an invariant face representation obtains across visual angles regardless of facial shape? **Taking stock of premechanistic insights** Neural correlates, causal boundaries, and causal pathways offer useful information for the task of mechanistic modeling. For example, a long-standing result in neuroscience is that the Fusiform Face Area (FFA) correlates with face processing (Kanwisher et al., 1997), with strong evidence for its causal relevance (Parvizi et al., 2012). To develop a mechanistic model of face processing, we need to find specific parts that implement specific algorithms of face processing. The conclusion that the computation’s parts and algorithms are embedded in the FFA invites an exploration of what is unique about that piece of cortex, and it also tells us where to test algorithmic and implementational hypotheses that arise from our model. **Asking mechanistic questions** Building on the previous point, we can ask a cluster of questions to jump-start our modeling efforts. Such questions include: • What algorithms are at play in face processing? Specifically, what are the relevant representations that it receives, and what sort of transformations are performed over it to derive different outputs? • What physical parts in the FFA implement the algorithms? Is the work carried out by local circuits, distributed networks, specific types of cells, classes of neurotransmitters, molecular processes inside cells, or something else? What are the parts that would do the work in a mechanistic model? • How do these parts systematically interact to result in face representations as opposed to some other computation, such as the computations of neighboring areas? • Is there something about FFA’s design that naturally instantiates certain algorithms? For example, is there a special columnar architecture, projection scheme (e.g., recurrence), canonical circuit layout, tuning curve profile, or temporal or spatial coding scheme that helps explain why specifically face representations obtain? • How does the FFA perform one computation (e.g., processing a round face) versus another (e.g., processing an oval face)? How do the activation or storage patterns differ in both cases, and why does each pattern result in the associated percept?

A necessary condition for capturing neural mechanisms of cognition is to explain what algorithms they implement. Through computational modeling efforts, we can explore what algorithms can achieve the computation at hand, fulfilling a criterion that is neglected in the premechanistic stage (Guest & Martin, 2021). Such algorithmic models must make specific hardware commitments, transforming them from free-floating models into mechanistic models. In practice, this project is a two-way street, involving the dynamic interplay between modeling and experimentation. From one direction, algorithmic models can generate hypotheses on what parts in the brain can plausibly implement the computations at hand. For example, if our algorithmic-level explanation involves updates to previous representations, this might point to recurrent connections in the brain. From the other direction, the parts we know to exist in our approximate causal boundary might have distinct features that naturally lend themselves to instantiating specific algorithms, which we can explore in our modeling efforts (see Box 1 for some examples). Through this back-and-forth process, we can generate hypotheses on what algorithms and hardware are at work in mechanistic boundaries. These can be put to the test, allowing us to gradually discard possible models to derive explanations of cognition in the brain. Although deep problems remain—such as the fact that there will always be a large space of mechanistic models that can account for the same behavior (Marder, 2011)—our metric of understanding tells us whether we are moving in the right direction. If our models allow us to programmatically alter computation by intervening on aspects of our explanans, or to predict what specific computation obtains across inputs, then this suggests we are reaching our explanatory aims.

In short, the premechanism stage supports mechanistic modeling because it provides contextual information that can be used to draw inspiration for algorithms and hardware specification while offering spatiotemporal constraints on where to validate our explanans—but it does not need to be completed for mechanistic modeling to proceed. By working on mechanistic models right now, we can supply our landscape of brain-to-cognition relations with explanations as to why different computations obtain across different situations.

## What Neural Mechanisms Do Not Need to Be

A neural mechanism of cognition is a series of physical parts in the brain that interact in systematic ways to implement algorithms that result in computation, and describing them requires an adequate account of Marr’s levels. To some, this perspective may appear restrictive, evoking a sense that little in the brain can meet these demands. To defend against this potential criticism, it may be helpful to highlight several properties that mechanisms do not necessarily need to have, hinting at the heterogeneity of mechanism.

Typically, when we think of a mechanism, we think of lawlike operations in which activation of one part necessarily causes an interaction with another—such as when a gear turns its neighbor to change the arms on a watch. This analogy works for relatively deterministic systems like RAM and DNA, but not for the highly stochastic brain. The brain does not work like clockwork—there is randomness everywhere (Deco et al., 2009; Rolls & Deco, 2010). To give one elementary example, the release of neurotransmitter vesicles from cells occurs over probability distributions—and this principle likely holds for any mechanism to be found in the brain.

This adds a layer of difficulty in the search for neural mechanisms of cognition, and it partially explains why brain-based predictions of any kind are imperfect. Nevertheless, the presence of randomness in any system does not dissolve mechanism (Craver, 2007; Craver & Tabery, 2015), nor does it mean that describing them becomes an insurmountable task. To see why this is so, imagine we inject RAM and DNA with ample randomness. This would have sweeping effects on computation, but it does not turn mechanism into nonmechanism because there are still design principles at work that govern the interaction between system parts. Similarly, if we take a machine that works on interacting gears, and we shave the ridges to curve slightly—causing each gear to turn its neighbors only sometimes—this does not dismantle the core mechanistic architecture.^7^

Another common intuition about mechanisms is that they are about what happens at the level of small parts in the brain. This is exemplified by the cherished catchphrase *underlying neural mechanisms*, which, if consistently used, points ever downward. But a special status of the microscale does not follow from either the philosophical conception of mechanism or from Marr’s levels (Krakauer et al., 2017). Certainly, any mechanism can be further decomposed into smaller mechanisms (Love, 2021). For example, a car realizes driving by the organized interaction of its engine, drivetrain, steering wheel, and brakes, but each part of this mechanism has its own mechanisms, which in turn have their own (Bechtel & Richardson, 1993). Nevertheless, a macroscale neural mechanism is valid in its own right, even if there is a bottoming-out process to be pursued where—for example—the biophysical mechanisms underlying neuronal activation are specified as well (Machamer et al., 2000). Indeed, the previously discussed model of visual decision-making operates at the scale of neurons and above but meets the high bar of mechanism. And to cite another example, Hasselmo et al. (2002) specify how subregions of the hippocampus systematically interact to promote either memory encoding or retrieval.

A final point concerns the spatial organization of mechanistic parts. At first blush, it might seem that a mechanistic view presupposes that computation is modular rather than distributed. Indeed, most covered models involve anatomical regions and local neuronal ensembles, in which the computational nuts and bolts are in each other’s vicinity. However, in cases in which highly distributed networks are the functional unit, nothing essentially changes. Whether we are dealing with a confined circuit or a network that is sparsely laid out across the brain, variation in computation will have to do with a difference in the brain’s design principles operating throughout physical parts—and the point stands that an adequate mechanistic explanation must derive these.^8^

In the next segment of this contribution, I will discuss what needs to be considered once a neural mechanism of cognition has been characterized. After that, I will enumerate some limitations and end with concluding remarks.

## The Scope of Neural Mechanisms

In cognitive neuroscience, statistical reliability is taken as the arbiter of what makes or breaks a putative neural mechanism of cognition. When a hypothesis that tests a neural mechanism is consistently left unrejected, we gain confidence in the mechanism, and we allow it to figure in theories of the cognitive brain. But it is one thing to establish that a neural mechanism of cognition exists, and it is another to establish how big a role it plays in cognition. This raises the question of how the scope of a candidate mechanism is currently estimated by the field.

It seems that the scope of mechanism is mostly estimated by conceptual analysis, through which we consider how large a conceptual gap is filled by a putative mechanism. Does it explain large swathes of how a cognitive function (such as decision-making) is implemented by the brain? Or does it offer more parochial answers, such as how reward is coded (which is only a subcomponent of decision-making)? In short, it seems a putative neural mechanism is taken to figure centrally or peripherally in explanations of cognition in the brain through a process of conceptual analysis (which, to be sure, is usually implicit).

In this section, I argue that effect size should be an additional consideration in our process of estimating explanatory scope (Funder & Ozer, 2019). Specifically, how much variance of the computation at hand—indexed by cognitive tasks, overt behavior, or other dependent variables—is accounted for by experimental manipulations on mechanistic parts? This is different from effect size on the level of neural measurement, which (for example) concerns the fidelity of neuroimaging components, such as blood-oxygen levels or the amplitude of ERPs. Naturally, tracking explained variance in neural signals can inform us whether we are targeting our mechanisms as intended, but it does not get at the heart of things. Rather, the key question is this: To what extent do manipulations that target the contents of a mechanistic model (the explanans) have consequences for the computations of cognition (the explanandum)? Let me take the previous mechanistic model to illustrate why teasing out effect size in this way can inform us of explanatory scope.

**Fig. 4.**
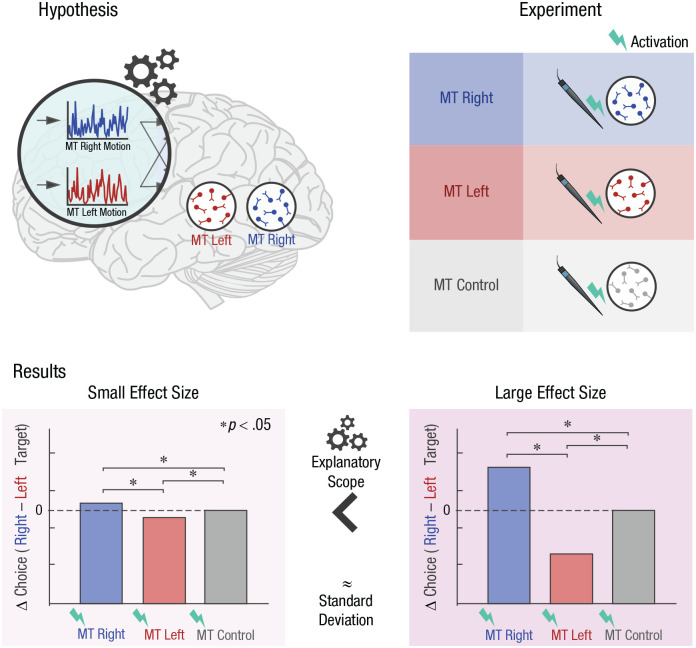
Effect size and the explanatory scope of neural mechanisms. As recapped in (a), the mechanistic model by Mazurek et al. (2003) assumes that one group of neurons in extrastriate region MT represents sensory evidence toward leftward motion, with another group representing rightward motion. If that is true, then decision-making is expected to change as a function of which group we activate. In a hypothetical experiment (b), we may use intracortical microstimulation to independently activate the two neuron groups. We may also include a control condition in which we stimulate a group of neurons thought not to represent motion direction (to further eliminate potential brain stimulation confounds). Our dependent variable might be the difference in the number of trials in which left or right targets are chosen. This difference yields an index of visual decision-making. If the hypothetical results (c) warrant a rejection of the null hypothesis, this provides grounds to support part of our mechanistic model. Crucially, effect size—which is usually neglected in cognitive neuroscience—provides information about explanatory scope. All else equal, small effect sizes (left) do not justify the interpretation that a central or fundamental mechanism is being tapped into. On the other hand, large effect sizes (right) justify hypotheses that situate the mechanism more ambitiously. It is important to note that effect-size estimation on the single-study level is uncertain, serving as a stepping stone for multistudy approaches (see main text). Note that this fictional experiment serves to illustrate a point on effect size and is not intended to represent an optimized design to comprehensively test the mechanistic model. MT = middle temporal area.

Imagine a hypothetical experiment that tests part of our putative neural mechanism of visual decision-making (Fig. 4). The model posits that one group of neurons in area MT code sensory evidence for leftward motion, while another group codes rightward motion (Fig. 4a). Now, we may build an experiment where, across conditions, we independently manipulate the activity for each group of neurons (using, e.g., microstimulation), perhaps adding a condition in which we target a control group of neurons thought to code for neither direction (Fig. 4b). If our mechanistic model is correct, we expect visual decision-making—indexed here as the difference between left and right target choice—to change depending on which neurons we stimulate.^9^

For this individual study, the kinds of interpretations that are justified toward the mechanistic model from which our hypothesis originated depend on obtained effect sizes—not just our conceptual analysis of the modeled mechanism (Fig. 4c). Namely, if we observe a statistically significant result but a small effect size, it would be unjustified to speculate that we are tapping into a central mechanism of visual decision-making—the data speak against this interpretation (Fig. 4c, left). Now contrast this with a scenario where we find that our model explains ample variance in target choice. In this case, we are empirically justified to raise more ambitious hypotheses for future studies to pursue.

With that said, effect sizes derived from single studies offer only uncertain estimates of the explanatory scope of a mechanism. After all, there are numerous reasons why a neural mechanism could fail to leave marked footprints on the cognitive-behavioral level in a single experiment. It could be overshadowed by more powerful processes, its effects could accumulate over a duration beyond what is measured, the mechanism could work differently across participants, or the experimental manipulation could fail to target enough mechanistic parts and operations—basically, all the same reasons why a neural mechanism’s effects may fail to be reliably detected in the first place.

Nevertheless, the basic point—that effect size is an important consideration in cognitive neuroscience—persists. Theoretical neuroscience involves taking neural mechanisms collected across research programs and figuring out how and where each of them fits in holistic and systematic explanations of the cognitive brain. And if mechanism A consistently accounts for more cognitive variance than mechanism B across studies, then this is a type of evidence informative toward this end.^10^ Specifically, by rank-ordering mechanisms in terms of explained variance, our decision process on what fits in the center of our theories becomes more objective, as we avoid placing all of our eggs in the basket of our own potentially flawed thinking. At present, however, effect size is rarely considered or explicitly communicated in cognitive neuroscience, which instead focuses on *p* values in its statistical reporting (which does not address explanatory scope; Sullivan & Feinn, 2012).

How would tackling effect size work in practice? An approach well-trodden in other fields is meta-analysis. By systematically aggregating results across studies and performing statistical analyses over them, the effect size of different neural mechanisms can be approximated and compared. This approach is bolstered by efforts to systematically organize neuroscience data (Akil et al., 2011; Markiewicz et al., 2021). On a separate track, the rapid growth of collective research efforts comes with unique opportunities for effect-size estimation. Multicenter initiatives make it possible to systematically explore parameter spaces, thereby getting a handle on factors that distort true effect sizes (Kandel et al., 2013). And in adversarial collaborations, neural mechanisms with a history of empirical success are pitted against each other in the same cognitive context to see which best accommodates the data (Cowan et al., 2020; Melloni et al., 2021). A further advantage of high-resource efforts like these is that they help disentangle the individual contribution of several correlated mechanisms, so that we do not confuse one explanation for another or wrongly estimate our effects (Tosh et al., 2022).

In summary, by factoring in how much cognitive variance is explained by a putative mechanism, we are giving nature an extra seat at the table, so to speak. Whereas conceptual inquiry offers deductively derived insights, effect size provides a data-driven estimation of explanatory scope that matters as well. It should be noted that there are other underutilized approaches to estimating explanatory scope besides estimating effect size. Most notably, one can address the question from an algorithmic angle, leaving brain experiments aside momentarily to pursue higher-level analyses (Levenstein et al., 2023). By modeling the algorithms assumed to be at play in different neural mechanisms, it may be found that one model manifests a wider range of computations that humans are known to perform, or it may be observed that one model strikes a particularly fine balance between accuracy and parsimony (Vandekerckhove et al., 2015) or some other epistemic virtue that gives it a leg up on other mechanistic hypotheses.

## A Few Considerations

What are some assumptions and limitations of the view presented? First and foremost, this work is deeply tied up with Marr’s three-level framework: The implementational, algorithmic, and computational levels are adopted as binoculars through which to understand the mind and brain. This means that if any of the levels are flawed or incomplete when put together then so too are the scientific explanations that they yield. A rich body of literature is dedicated to Marr’s epistemology and its limitations (Peebles & Cooper, 2015), which includes discussions on what each level means or should mean (Hardcastle & Hardcastle, 2015), suggestions on what level might be missing from the picture (Poggio, 2012), and critiques of levels of analyses as a meta theoretical method in the first place (Danks, 2013). Although a good many of the arguments presented in this contribution will not collapse even if one rejects Marr’s levels, some will, and most of them will be affected in one way or another. Therefore, criticisms of Marr’s framework carry over to the perspective offered here.

Second, still concerning assumptions, one might question whether the project of mapping algorithms to parts of the brain and that of mapping computations onto algorithms can be done reliably. To touch on this some more, *multiple realizability* is continually looming on the horizon, especially in a framework that seeks to have different levels converge. Just as countless computer programs can find the maximum value in a matrix, and just as summing algorithms can be realized by devices that vary in physical makeup, so too are the parts, algorithms, and computations of the brain underdetermined by each other. This leaves us in a state of uncertainty as to whether our mechanistic models reflect the brain’s operations even as we gather what-if-things-had-been-different answers. Perhaps the way to deal with this is through a project of gradual expansion. After a mechanistic model has been found to account for computations in one experimental context, it can be brought to bear on another—ideally one that ups the ecological validity. With appropriate revisions or full-on redevelopments, we might build increasingly encompassing mechanistic models of cognition and iterate toward truth.

Third, in terms of reach itself, there is more to understanding cognition than mapping out every mechanism there is to be found in the brain. For one, the present proposal has said little about the computational level that sets our explananda: Except for a recommendation in Box 1, I have mostly adopted it as fixed, viewing it as ready to work with. But spelling out what cognition consists of, how various computations interlink, and why the structure of the world leads us to process information in the distinct ways we do (Bechtel & Shagrir, 2015) are all important undertakings because they delineate, contextualize, and indeed fill up our target explananda. Every level offers unique constraints that narrow the possible answers on other levels, and the computational level is no exception.

## Concluding Remarks: Toward Unification

This contribution echoes the sentiment that linking cognition to segments of the brain does not give adequate explanations for cognitive neuroscience. Mechanism is needed, and that much is agreed upon. But how the term is used varies substantially within and across research programs. Although variation in language use does not have to be a problem by itself, it does point to a lack of agreement on what properties a neural mechanism has and how we should go about looking for them. This runs the risk of hindering convergence at the theoretical level, where fruits harvested across programs need to be integrated. Less allegorically, if each program employs a different format of scientific explanation, a unified cognitive neuroscience is hard to reach.

In this contribution, I have argued that combining Marr’s framework with recent philosophical work on mechanisms in science offers a unifying lens through which we can formalize the goal of explaining how the brain realizes cognition. The short version is that we need a specification of the brain’s design principles and the parts that realize them to produce computation. Such an account leaves significant heterogeneity on what in the brain can make for a mechanism while offering clear limits on what cannot.

For example, neural mechanisms can involve parts of the brain below the scale of neurons or above it, and they can either be huddled together or spread out in space. All neural mechanisms are causal, but not all causal relations in the brain are neural mechanisms. Also, neural mechanisms do not need to be deterministic—although the brain is stochastic, there is still an underlying mechanistic architecture of cognition to be carved out by the joints. Finally, to meet the bar of neural mechanism, parts of the brain must be subject to computing algorithms rather than simply activate in an algorithmically impoverished sequence, such as in a causal pathway.

Cross-program unification involves building a mosaic of all established findings. I have argued that in building this mosaic, our decision process on what fits in the center and periphery can benefit from considering the explained variance of candidate neural mechanisms toward the explanandum. Finally, mechanistic understanding can be quantified by the degree to which formulated algorithms applied to brain data help predict or alter cognition—testing whether description maps onto reality. Together, hopefully these reflections serve to invigorate a mechanistic approach to cognitive neuroscience that cuts across research programs. To end with the obligatory mantra, then, more research is needed to understand the neural mechanisms of cognition.

## Transparency

*Action Editor:* Rogier Kievit

*Editor:* Rogier Kievit

## Notes

1. There are different accounts of what computational-level descriptions must consist of. For an overview of these accounts, see the work of Bechtel and Shagrir (2015), Rescorla (2020), and Shagrir and Bechtel (2017, section 6.2).

2. Marr’s three-level framework is a way to *analyze* systems that have mechanisms (an epistemological project), with mechanism itself *being* the organized interaction between parts that results in a phenomenon (a metaphysical claim). My argument is that these considerations together serve to refocus work in search of neural mechanisms, not that Marr’s framework and levels of mechanism collapse into each other (Piccinini & Craver, 2011).

3. We can even exploit our mechanistic understanding of DNA to build efficient memory systems (Goldman et al., 2013), which allow us, for example, to store Shakespeare’s sonnets (Yong, 2013).

4. Caution is warranted with this type of inference, because the finding that cognition changes as a function of causal interventions can be explained by a plethora of confounders. For alternative explanations in brain stimulation, see Bergmann and Hartwigsen (2021), Schutter and Hortensius (2010), and van Bree et al. (2019). For alternative explanations in lesion studies, see Vaidya et al. (2019).

5. That is to say, the stimulation has a local effect that impairs the intraregion computations directly while exerting only indirect influences on downstream regions by altering their input as a consequence of altered functioning upstream.

6. Models that meet the necessary criteria for mechanism may nevertheless vary substantially in richness. For example, a basic model might describe neuronal ensembles that implement evidence accumulation up to a threshold. Then subsequent research in the mechanism stage might enrich the model with details on where the threshold lies, the dynamics by which evidence accumulation unfolds, factors that explain how stochastic the process is, or any other detail that further explains what computations obtain across circumstances.

7. The question addressed in this paragraph is whether the concept of mechanism holds up in random systems. This should not be confused with the question of whether randomness itself can figure in mechanism (which it can, see Harris & Wolpert, 1998, for a seminal example).

8. With that said, Pessoa (2023) summarized some reflections on distributed computation, interactional complexity, and functional decomposition that could have implications for the view presented here.

9. Note again that in our effect-size estimation, we are fixating on cognition rather than on neural signals. Indeed, if we succeed at stimulating the left- and right-coding ensembles focally without having stimulation shunt to nearby areas, the locally induced action potentials are unlikely to exert much of a footprint in neuroimaging signals. Yet if the targeted neurons are where motion direction is coded, the effects of the experimental manipulation on behavior will be large. In other words, variance on the neural signal and cognitive level may dissociate, and what matters for assaying scope is the latter, because it indexes the explanandum.

10. Direct comparisons do not require external reference frames, so even if difficult questions (like what constitutes a small or large effect size) remain, this need not prevent the project of comparing mechanisms from going forward.
